# Editorial: Epigenetics and neurodevelopment in psychiatry

**DOI:** 10.3389/fgene.2024.1473521

**Published:** 2024-08-19

**Authors:** Jacob Peedicayil

**Affiliations:** Department of Pharmacology and Clinical Pharmacology, Christian Medical College, Vellore, India

**Keywords:** epigenetic, neurodevelopment, psychiatric disorders, psychiatry, epigenetc

Epigenetics is a term that was coined by combining the words epigenesis (embryonic development) and genetics. In this light, epigenetics plays a major role in development of body tissues and is especially important for the development of the brain, where mutations in genes encoding enzymes involved in epigenetic mechanisms of gene expression have been shown to result in neurodevelopmental disorders (Keverne and Binder, 2020). For instance, mutations in the gene encoding methyl-CpG binding protein (*MECP2*), a protein needed for neuron function, results in Rett syndrome (Keverne and Binder, 2020). Kleefstra syndrome, a rare genetic disorder where there is intellectual disability, is caused by haploinsufficiency of the gene encoding euchromatic histone lysine methyltransferase 1(EHMT1) (Colijn et al., 2023).

During the development of an individual’s brain, the three major types of brain cells, neurons and glial cells (astrocytes and oligodendrocytes), form from neural stem cells (cells that are able to self-renew and differentiate into the three major brain cell types) (Peedicayil and Grayson, 2023). Epigenetic mechanisms play a key role in controlling gene expression in this process by regulating changes in chromatin structure (Peedicayil and Grayson, 2023). Epigenetic mechanisms also play a major role in the change of the foetal brain into the adult brain, being involved in various processes including memory, synaptic transmission, neuronal plasticity, cognition and behaviour, neuroendocrinology and neuroimmunology (Peedicayil and Grayson, 2023). The human brain continues to develop throughout life (Ventriglio et al., 2016). Chronological aging is often accompanied by major structural and functional changes in the brain (Ventriglio et al., 2016). Hence, the sub-speciality of psychiatry, developmental psychiatry, covers the lifespan of an individual and involves epigenetic mechanisms across the lifespan (Ventriglio et al., 2016; Peedicayil, 2017).

In the light of the above-mentioned critical functions of epigenetics in the brain, it is no surprise that dysregulated epigenetic mechanisms play an important role in psychiatric disorders. This Research Topic of *Frontiers*, entitled Epigenetics and Neurodevelopment in Psychiatry, is devoted to the study of the role of epigenetics in psychiatric disorders. Since genetics and epigenetics are closely related to each other, and are sometimes considered to be two sides of the same coin (You and Jones, 2012), this issue treats the topic of epigenetics broadly, and also includes articles on the genetics of the brain.

The gene encoding LIM-kinase1 in the *Drosophilia melanogaster* mutant agn^ts3^ is a hotspot for chromosome breaks, ectopic contacts, under-replication, and recombination. agn^ts3^ is a simple model for Williams-Beuren syndrome, a rare neurodevelopmental disorder whose symptoms include cognitive impairment. Savvateeva-Popova et al. found that the overall expression of microRNAs (miRNAs) is markedly decreased in agn^ts3^. The phenotypes of human imprinted neurogenetic disorders can be considered to be extreme variations of normal human phenotypes (Salminen et al.). Prader-Willi syndrome (PWS), is a neurogenetic disorder that involves imprinting. Salminen et al. phenotyped a group of normal individuals for psychological and behavioural traits found in PWS. The authors investigated the pattern and extent of co-variation (correlated variation of two or more variables) shown for these traits using principle component analysis, a machine learning method for simplifying a large amount of data into a smaller set while maintaining significant patterns and trends. The authors found that the diverse phenotypes of PWS reflect extremes of co-variation in the normal population. Shiota et al. used case-control next-generation sequencing combined with psychological assessments on Japanese children with autism spectrum disorder (ASD). The authors explored previously found ASD-associated genes and determined if they are associated with behavioural traits. The authors found that the investigated genes are necessary for the development of ASD. Wu et al. attempted to find the incidence rate of temporomandibular joint disorders in patients with psychiatric disorders. The authors used data from the Psychiatric Genomics Consortium and the FinnGen databases. Using a two-sample Mendelian randomization approach, the authors show that there is an elevated risk for temporomandibular disorder in patients with attention-deficit hyperactivity disorder (ADHD).
